# Daphnetin alleviates unexplained recurrent spontaneous abortion by regulating the NR4A1/BACH2 axis in mice

**DOI:** 10.1186/s40659-025-00658-7

**Published:** 2025-12-29

**Authors:** Zhiqin Zhang, Jun Tan, Xingwu Wu, Xin Li, Peipei Liu, Liyun Cao, Shenggen Long

**Affiliations:** 1 Reproductive Medicine Center, Jiangxi Maternal and Child Health Hospital, Nanchang, 330006 Jiangxi Province China; 2 Jiangxi Key Laboratory of Reproductive Health, Nanchang, 330006 Jiangxi Province China; 3 JXHC Key Laboratory of Fertility Preservation, Jiangxi Maternal and Child Health Hospital, Nanchang, 330006 Jiangxi Province China; 4 Department of Gynecological Oncology, Jiangxi Maternal and Child Health Hospital, No. 318, Bayi Avenue, Nanchang, 330006 Jiangxi Province China

**Keywords:** Daphnetin, Unexplained recurrent spontaneous abortion, Th17/Treg cell homeostasis, NR4A1, BACH2

## Abstract

**Background:**

Daphnetin has demonstrated various pharmacological activities. The current study evaluated the potential of daphnetin in alleviating unexplained recurrent spontaneous abortion (URSA) and explored underlying mechanisms.

**Methods:**

Mice with URSA were gavaged with 1 mg/kg, 10 mg/kg, and 20 mg/kg of daphnetin, or infected with adeno-associated viruses harboring knockdown of NR4A1 or overexpression of BACH2 before modeling. Human peripheral blood T lymphocytes were induced into CD4^+^ T cells, followed by lentivirus infection and daphnetin treatment. The influence of daphnetin on CD4^+^ T cell viability and Treg and Th17 cell differentiation in cells was analyzed. The concentrations of Treg cells-associated cytokines (TGF-β, IL-10) and Th17 cells-associated cytokines (IL-17, IL-23) in the supernatants of CD4^+^ T cells were assessed. The regulation of NR4A1 on BACH2 was analyzed by ChIP and dual-luciferase assays.

**Results:**

Daphnetin resulted in fewer immature, resorbed, or dead embryos in mice with URSA, with the most pronounced therapeutic effect of 10 mg/kg. Daphnetin attenuated decidual hemorrhage, with a gain in the percentage/number of Treg cells and a loss of the percentage/number of Th17 cells in the spleen and decidual tissues. Daphnetin enhanced the expression of FoxP3, TGF-β, and IL-10, and suppressed the expression of RORγt, IL-17, IL-23, and the contents of TNF-α, IL-6, and IL-1β in CD4^+^ T cells. Overexpression of BACH2 further alleviated URSA deterioration caused by NR4A1 knockdown. Daphnetin mediated the transcriptional activation of BACH2 by upregulating NR4A1.

**Conclusions:**

Upregulation of NR4A1 by daphnetin mediates BACH2 transcription and Th17/Treg cell homeostasis to improve URSA.

## Introduction

Recurrent spontaneous abortion (RSA) refers to the occurrence of two or more spontaneous abortions before or during the early stages of gestation [1]. It is a devastating reproductive health burden that disturbs approximately 5% of couples trying to conceive in the world, and the number of cases with unexplained etiology (URSA) reaches 50% [2]. The immunological perspective in URSA has gained extensive attention, and a successful pregnancy requires a transition to an immune environment favorable for embryo survival at the maternal–fetal interface [3]. The helper T (Th)17/regulatory T-cell (Treg) cell paradigm has emerged as a novel subpopulation of CD4^+^ T cells and helps to explain the pathology of RSA [4]. Treg cells, which express IL-10 and TGF‐β, play a key role in pregnancy tolerance, while Th17 cells are accountable for pregnancy loss and autoimmunity by releasing cytokines such as IL-17 and IL-22 [5]. Therefore, maintaining Th17/Treg cell homeostasis might hold innovative significance in URSA.

As an increasingly well-known derivative of coumarin, daphnetin has demonstrated distinct pharmacological activities, including anti-inflammatory properties, anti-tumor, and anti-autoimmune diseases [6]. Interestingly, daphnetin inhibited the production of IL-17A in developing Th17 cells under bleomycin-induced pulmonary fibrosis [7]. However, the potential of daphnetin in regulating Th17/Treg cell homeostasis in URSA has not been revealed. The nuclear receptor subfamily 4A (NR4A) family encodes orphan nuclear hormone receptors with transcriptional activity and has been implicated in different aspects of lymphocyte development, tolerance, as well as function, including roles in Treg and exhaustion [8]. For instance, purified CD4^+^ T cells from naïve NR4A1^−/−^ mice exhibited enhanced Th17 differentiation capacity [9]. Moreover, NR4A1 (also known as Nur77) expression was reduced by 60% in the endometrium of the patients with recurrent implantation failure, and NR4A1 overexpression reversed the KLF12-mediated inhibition of decidual prolactin (PRL) expression, decidual transformation, and blastocyst-like spheroid expansion [10], which suggested that restoration of NR4A1 might be a target for pregnancy complications. In this research, we identified NR4A1 as a direct target of daphnetin and BTB Domain and CNC Homolog 2 (BACH2) as a target of NR4A1. BACH2 plays a vital role in maintaining the suppressive function of transforming growth factor-beta (TGF-β)-derived human forkhead box protein P3 (FOXP3)^+^ Treg [11]. Furthermore, BACH2 has been shown to impede Th17 differentiation by repressing the expression of lineage-associated genes, including AHR [12]. As a consequence, the regulation of daphnetin on the immune homeostasis of Treg/Th17 cells through the activation of the NR4A1/BACH2 axis during URSA was investigated in this study.

## Materials and methods

### Establishment and treatment of the URSA mouse model

Male BALB/c mice (19–25 g) and male DBA/2 mice (22–24 g) at 8–10 weeks were obtained from Vital River (Beijing, China), and female CBA/J mice (16–22 g) at 6–8 weeks were procured from HFK (Beijing, China). All mice were maintained with a 12 h light/12 h dark cycle at a constant temperature (26 °C) under standardized conditions. The animal experimental protocol was approved by the Medical and Experimental Animal Ethics Committee of Jiangxi Maternal and Child Health Hospital.

After an acclimatization period, female CBA/J mice were divided into eight groups. Female CBA/J mice (n = 10) mated with male BALB/c mice (n = 10) were allocated to the normal group. URSA (n = 10), URSA + normal saline (NS) (n = 10), URSA + Daphnetin (n = 30 for different doses), short hairpin RNA (sh)-negative control (NC) + URSA + Daphnetin (n = 10), sh-NR4A1 + URSA + Daphnetin (n = 10), sh-NR4A1 + overexpression (oe)-NC + URSA + Daphnetin (n = 10), and sh-NR4A1 + oe-BACH2 + URSA + Daphnetin (n = 10) groups were constructed by mating female CBA/J mice with male DBA/2 mice. Detection of a positive vaginal plug indicated the onset of pregnancy (day 0.5 of pregnancy). Subsequently, 1 mg/kg, 10 mg/kg, and 20 mg/kg daphnetin (HY-N0281, MedChemExpress, Monmouth Junction, NJ, USA) were administered by gavage from 0.5 days post-pregnancy for 13 d [7].

Female CBA/J mice were infected with 100 μL of adeno-associated viruses harboring sh-NR4A1, oe-BACH2, sh-NC, or oe-NC (both viral titers 2 × 10^11^ GC /mL) via tail vein injection and mated with DBA/2 males 2 weeks later. At 14.5 days of gestation, female mice were euthanized by sodium pentobarbital (*i.p.*, 150 mg/kg). The uterus, embryo, and decidual tissues were removed and photographed, and the decidual tissues were preserved for subsequent experiments [13]. The site of uterine implantation showed a small, dark residue or complete absence, indicating embryo resorption. Embryo resorption rate = number of embryos resorbed/total number of embryos × 100%. All histological evaluations were performed by an investigator who was unaware of the group assignments.

### Histological and immunohistochemical analysis

Mouse decidual tissues were fixed using 4% paraformaldehyde, paraffin-embedded, and sectioned at 4-μm. The sections were deparaffinized with xylene (10 min) and rehydrated using ethanol (100%, 5 min; 90%, 2 min; 80%, 2 min; 70%, 2 min) and distilled water (2 min). After staining with hematoxylin staining solution (C0105M, Beyotime, Shanghai, China) for 10 min, the sections were rinsed with tap water, stained with eosin staining solution for 1 min, and washed with 70% ethanol. After staining with hematoxylin–eosin (HE), the sections were dehydrated using an increased ethanol gradient and cleared with xylene. Slides mounted with neutral gum were subsequently viewed under a microscope, and the stained sections were captured.

Paraffin-embedded decidual Sections (4-μm-thick) were deparaffinized, rehydrated, antigenically repaired using citrate buffer (pH = 6.0), and treated with 3% H_2_O_2_. Nonspecific binding was blocked with 5% normal goat serum. The sections were probed with primary antibodies to IGFBP-1 (1/1000, PA5-79447, Thermo Fisher Scientific Inc., Waltham, MA, USA), PRL (1/8000, ab183967, Abcam, Cambridge, UK), NR4A1 (1/50, PA5-34033, Thermo Fisher Scientific), and BACH2 (1/100, PA5-100792, Thermo Fisher Scientific) overnight at 4 °C and with HRP-conjugated goat anti-rabbit secondary antibody (1/10000, ab205718, Abcam) for 1 h at room temperature. The signal was developed using 3,3′-diaminobenzidine (34002, Thermo Fisher). The sections were counterstained with hematoxylin, sealed with neutral gum, and placed under the microscope. The percentage of positive cells was used to characterize the result of immunohistochemical analysis.

For immunofluorescence staining, decidual tissue sections were incubated with 5% normal goat serum. The sections were labeled using Alexa Fluor 488-conjugated anti-CD4 antibody (1:100, ab277275, Abcam) and eFluor 660-conjugated anti-IL-17A antibody (1:100, 50-7177-82, Thermo Fisher Scientific) at 4 °C in the dark overnight to detect Th17 cells (CD4^+^IL-17A^+^). To detect Tregs (CD25^+^FoxP3^+^), decidual tissue sections were incubated overnight at 4 °C with rabbit anti-CD25 (1:200, PA5-116978, Thermo Fisher Scientific) and mouse anti-FoxP3 (1:1000, ab253297, abcam). The following day, the sections were incubated with Alexa Fluor 488-conjugated goat anti-rabbit IgG (1:1000, ab150077, Abcam) and Alexa Fluor 647-conjugated goat anti-mouse IgG (1:1000, ab150115, Abcam) at room temperature in the dark for 1 h. After labeling cell nuclei with DAPI, the number of Th17 cells or Treg was counted under a fluorescence microscope.

### Cell culture

Human peripheral blood T lymphocytes (SNP-H285, Sunncell, Wuhan, Hubei, China) were suspended at 2 × 10^6^ cells/mL in RPMI-1640 complete medium (11875093, Thermo Fisher) supplemented with 100 μg/mL streptomycin, 100 U/mL penicillin, 2 mM glutamine, and 10% heat-inactivated FBS. CTS Dynabeads CD3/CD28 (40203D, Gibco, Carlsbad, CA, USA) was added for T cell expansion. The cultures were induced with 50 ng/mL PMA (BML-PE160-0001, Enzo Biochem, New York, NY, USA) plus 1 μg/mL ionomycin (ALX-450-007-M001, Enzo Biochem) for 5 h in the presence of 500 ng/mL monensin (ALX-380-026-M100, Enzo Biochem) to activate the cytokine-producing capacity of T cells (Model group). CD4^+^ T cells were isolated by the EasySep Human CD4^+^ T Cell Isolation Kit (#17952, STEMCELL Technologies, Vancouver, BC, Canada) and transferred to 5 mL sterile tubes for subsequent staining.

According to the experimental requirements, lentiviruses harboring sh-NR4A1 with puromycin resistance and/or oe-BACH2 with neomycin resistance (both with viral titers of 10^8^ TU/mL) and their negative controls (sh-NC, oe-NC) were mixed with 8 mg/mL polyethylene (TR-1003, Sigma-Aldrich Chemical Company, St Louis, MO, USA). The mixtures were supplemented to CD4^+^ T cells with RPMI-1640 to achieve 500 μL/well. After incubation at 37 °C for 5 h, 500 μL of RPMI-1640 complete medium was supplemented. The cells were centrifuged at 240 g for 30 min, and the medium was aspirated and refreshed with RPMI-1640. Puromycin (2 μg/mL) and/or neomycin (200 μg/mL) were added to the medium and screened for 72 h to obtain stably transfected cells. The transduced cells were collected for Daphnetin treatment.

### CCK-8

CD4^+^ T cells were seeded in 96-well plates and cultured for 24 h. The cells were treated with daphnetin at 0, 5, 10, 20, 40, and 80 μM for 48 h. To each well, 10 μL of CCK-8 solution (C0037, Beyotime) was added and incubated with 5% CO_2_ at 37 °C for 22 h. Subsequently, the OD value at 450 nm was read using a microplate reader.

### Flow cytometry

Mouse splenic lymphocytes were isolated and prepared as individual cells. The cells were labeled using the following monoclonal antibodies: phaeohydroxin-Cy7 (PE-Cy7)-coupled rat anti-mouse CD4 (563933, BD Biosciences, San Jose, CA, USA), FITC-coupled rat anti-mouse CD25 (558689, BD Biosciences), PE-coupled rat anti-mouse FoxP3 (563101, BD Biosciences), and FITC-coupled rat anti-mouse IL-17A (11–7177-81, Thermo Fisher). CD4-positive cells were isolated with an anti-CD4 antibody. CD4-positive cells were isolated with an anti-CD4 antibody and stained with an anti-CD25 monoclonal antibody, followed by fixation using Fix/Perm solution (554714, BD Biosciences), permeabilization, and staining with anti-FoxP3 antibody or anti-IL-17A.

CD4^+^ T cells were dispensed into test tubes and incubated with PE-Cy7-coupled mouse anti-human CD4 (25-0049-42, Thermo Fisher), mouse anti-human CD25 (11-0257-42, Thermo Fisher), PE-coupled mouse anti-human IL-17A (12-7178-42, Thermo Fisher), and PE-coupled mouse anti-human FoxP3 (12-4774-42, Thermo Fisher). For Treg staining, incubation was performed using antibodies to CD25 and FoxP3. For Th17 cells, cells were incubated using antibodies to CD4 and IL-17A. CytExpert software was used for analysis.

### ChIP

SimpleChIP Enzyme ChIP Kit (#9003, Cell Signaling Technologies, Beverly, MA, USA) was utilized. CD4^+^ T cells in 20 mL of medium were treated with 540 μL of 37% formaldehyde for 10 min at room temperature. After another 5-min incubation with 2 mL of 10 × glycine, the cells were centrifuged at 500×*g* for 5 min at 4 °C, and the precipitate was rinsed twice with 20 mL PBS. The cells were resuspended in 1 mL of ice-cold 1 × Buffer A + DTT + PIC, ice-bathed for 10 min, and detached with 0.5 μL of micrococcal nuclease at 37 °C for 20 min to a DNA length of about 150–900 bp. Chromatin sample (50 μL) was incubated with 100 μL of nuclease-free water, 6 μL of 5 M NaCl, and 2 μL of RNAse A for 30 min at 37 °C and with 2 μL of Protease K at 65 °C for 2 h. DNA was purified from the samples using a DNA purification centrifuge column. Antibodies against NR4A1 (1/500, NB100-56745SS, Novus Biological Inc., Littleton, CO, USA) were used to precipitate the NR4A1-bound chromatin, using rabbit IgG as a control. Then, 30 μL of protein G magnetic beads were added for a 2-h incubation at 4 °C. Chromatin was eluted from the antibody/protein G magnetic beads, and the DNA was de-crosslinked by incubation with 6 μL of 5 M NaCl and 2 μL of proteinase K for 2 h at 65 °C. DNA purification was performed, and qPCR amplification was performed by promoter-specific primers for BACH2.

### Luciferase assay

The BACH2 promoter sequence (chr6: 90296739–90297088) containing the NR4A1 binding sequence (CCGAATGTCAAC) was inserted into the Promoterless Firefly Luciferase Basic Vector (E6651, Promega Corporation, Madison, WI, USA). Subsequently, C4D^+^ T cells stably knocked down for NR4A1 were co-transfected with Promoter-Driven Control Renilla Luciferase Vectors (E6911, Promega) and firefly luciferase vectors containing promoter sequences for 48 h using Lipofectamine 3000 (L3000075, Thermo Fisher). Relative luciferase activity was assayed using the Dual-Luciferase Reporter Gene Assay Kit II (RG029M, Beyotime). Firefly luciferase activity was normalized against Renilla luciferase activity, which served as the transfection control.

### Measurement of cytokines by ELISA

The concentrations of Treg cells-associated cytokines TGF-β (BMS249-4, Thermo Fisher), IL-10 (88-7106-88, Thermo Fisher), Th17 cells-associated cytokines IL-17 (A356115, Thermo Fisher), IL-23 (88-7237-88, Thermo Fisher), as well as the pro-inflammatory cytokines TNF-α (ab181421, Abcam), IL-6 (ab178013, Abcam), and IL-1β (ab108865, Abcam) were evaluated in CD4^+^ T cell culture supernatants. The OD value was read at 450 nM.

### qPCR analysis

Total RNA was extracted from mouse decidual tissues and CD4^+^ T cells using TRIzol. Reverse transcription was performed using the PrimeScript RT reagent kit (RR037Q, Takara, Dalian, Liaoning, China) according to the manufacturer’s instructions. qPCR was performed using ChamQ Universal SYBR qPCR Master Mix (Q711, NanJing Vazyme Biotech Co., Ltd., Nanjing, China). GAPDH served as a housekeeping gene. The primer sequences are shown in Table 1. The relative expression was analyzed by the 2^−ΔΔCt^ method.

**Table 1:** Sequence of primers used to amplify target genes

Gene	Forward sequence (5’-3’)	Reverse sequence (5’-3’)
FoxP3 (mouse)	CCTGGTTGTGAGAAGGTCTTCG	TGCTCCAGAGACTGCACCACTT
FoxP3 (human)	GGCACAATGTCTCCTCCAGAGA	CAGATGAAGCCTTGGTCAGTGC
RORγt (mouse)	GTGGAGTTTGCCAAGCGGCTTT	CCTGCACATTCTGACTAGGACG
RORγt (human)	GAGGAAGTGACTGGCTACCAGA	GCACAATCTGGTCATTCTGGCAG
NR4A1 (mouse)	GTGCAGTCTGTGGTGACAATGC	CAGGCAGATGTACTTGGCGCTT
NR4A1 (human)	GGACAACGCTTCATGCCAGCAT	CCTTGTTAGCCAGGCAGATGTAC
BACH2 (human)	CTGCCGCAAAAGGAAACTGGAC	GGAAAGGCAGGAGAAGTTGTCC
GAPDH (mouse)	CATCACTGCCACCCAGAAGACTG	ATGCCAGTGAGCTTCCCGTTCAG
GAPDH (human)	GTCTCCTCTGACTTCAACAGCG	ACCACCCTGTTGCTGTAGCCAA

FoxP3, forkhead box protein P3; NR4A1, nuclear receptor subfamily 4immunitygroup A member 1; BACH2, BTB and CNC homolog 2; GAPDH, glyceraldehyde-3-phosphate dehydrogenase

### Western blot analyses

Total cellular fraction proteins were extracted from mouse decidual tissues and CD4^+^ T cells using RIPA lysis buffer (89901, Thermo Fisher). Nuclear and Cytoplasmic Protein Extraction Kit (P0028, Beyotime) was used to obtain cytoplasmic and nuclear fraction proteins from CD4^+^ T cells. The protein was separated utilizing 10% SDS-PAGE and electrically transferred to the PVDF transfer membrane (88518, Thermo Fisher). The PVDF membranes were blocked with 5% skimmed milk powder for 1 h and incubated with the primary antibodies to IGFBP-1 (1/500, PA5-79447, Thermo Fisher), PRL (1/1000, ab183967, Abcam), NR4A1 (1/1000, ab153914, Abcam), BACH2 (1/2000, PA5-100792, Thermo Fisher), GAPDH (1/2500, ab9485, Abcam), and Histone H3 (1/1000, ab1791, Abcam) overnight at 4 °C and with secondary antibody (1/10000, ab205718, Abcam) for 2 h at room temperature. The protein-band signal was detected with the ECL Chemiluminescent Substrate Reagent (WP20005, Thermo Fisher). Using ImageJ software, the gray values of the target protein blots were normalized against the internal control blots. GAPDH served as the internal control protein for the whole-cell fraction or cytoplasmic fraction, while Histone H3 acted as the internal control protein for the nuclear fraction.

### Protein stability test

CD4^+^ T cells were treated with 20 μM Daphnetin (or an equivalent volume of solvent DMSO) together with 10 μg/mL cycloheximide (CHX, S7418, Selleck, Shanghai, China) and harvested at designated time points (0 h, 2 h, 4 h, 8 h). Intracellular NR4A1 levels were determined using Western blot analysis and normalized based on NR4A1 protein expression at 0 h.

### Statistical analyses

GraphPad Prism software (ver. 10.4.2, GraphPad Software, Inc., San Diego, CA, USA) was used for data analysis. Data are presented as means ± SEM. Nonlinear regression was used to calculate the half-life of proteins. An unpaired t-test was used for comparison between the two groups, and a one-way/two-way ANOVA was performed using Tukey’s multiple comparison tests for multiple comparisons. The threshold for significance was set to *p* < 0.05.

## Results

### Daphnetin improves URSA in mice by maintaining the Th17/Treg balance

**Fig. 1:**
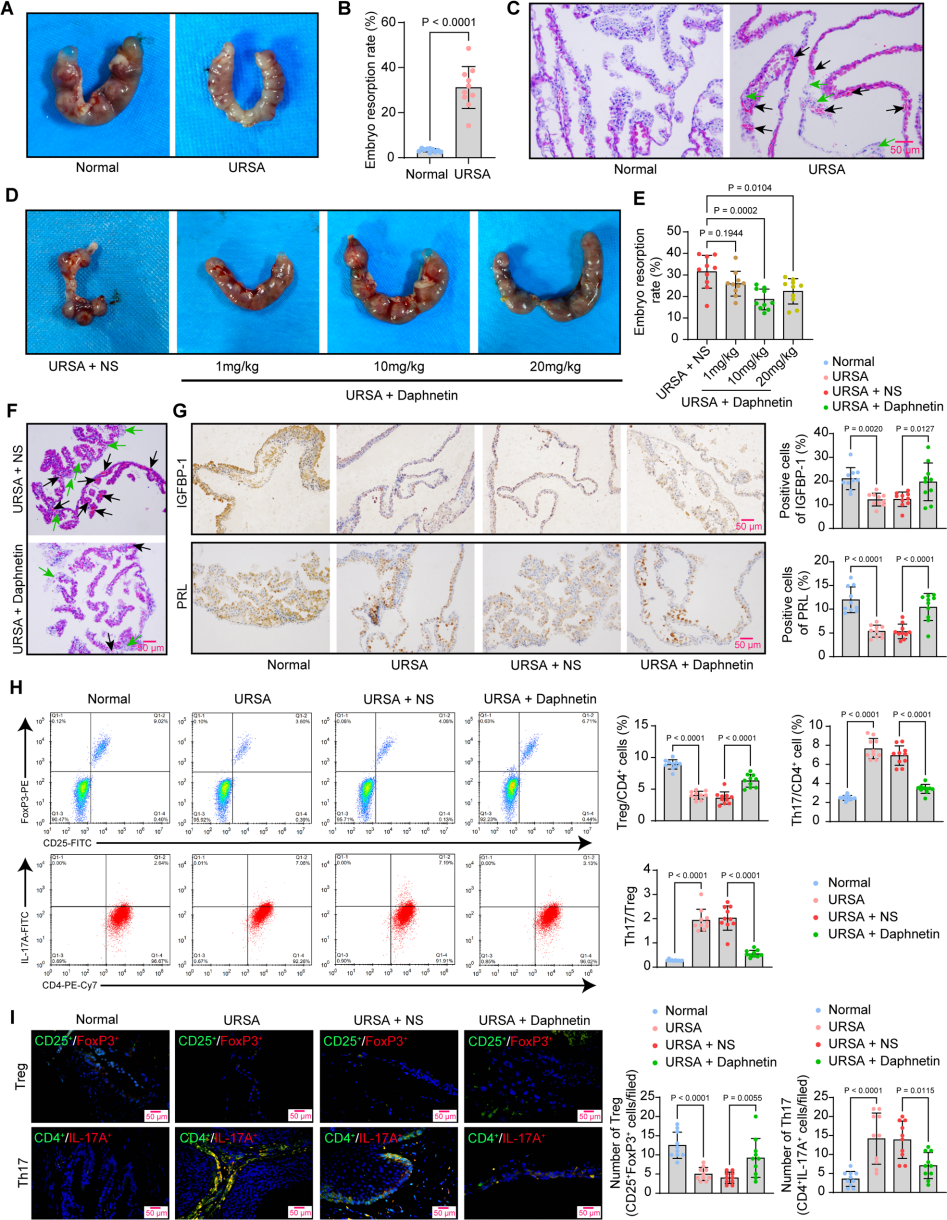
Daphnetin ameliorates URSA by regulating Th17/Treg balance. **A** Representative images showing the embryo resorption sites of normal and URSA mice on the 13th day of pregnancy in each group. **B** Embryo resorption rate in mice of the normal and URSA groups. **C** Morphologic changes (the black arrow indicates the site of hemorrhage, while the green arrow indicates the area of necrosis) in decidual tissues of normal and URSA mice were assessed by HE staining. **D** Representative images showing the embryo resorption sites of URSA mice treated with NS or daphnetin at different doses on the 13th day of pregnancy. **E** Embryo resorption rate in URSA mice treated with NS or daphnetin at different doses. **F** Morphologic changes (the black arrow indicates the site of hemorrhage, while the green arrow indicates the area of necrosis) in decidual tissues of URSA mice treated with NS or daphnetin were assessed by HE staining. **G** Positive cells of IGFBP-1 and PRL in decidual tissues of mice were detected by immunohistochemistry. **H** Treg and Th17 cell populations in the spleens of mice were analyzed using flow cytometry. **I** The number of Tregs (CD25^+^FoxP3 +) and Th17 cells (CD4^+^IL-17A^+^) infiltrating the decidual tissues of mice was assessed by immunofluorescence staining. Data is presented as mean ± SEM (n = 10). One-way ANOVA for EGHI, unpaired t-test for B

First, we constructed the URSA mouse model. In the normal group (CBA/J female mice mated with BALB/c male mice), almost no resorption and immature embryos were observed. However, in the URSA group (CBA/J female mice mated with DBA/2 mice), not all embryos were mature, and some were resorbed or died (Fig. 1A). The embryo resorption rate in the URSA group was significantly higher than that in the normal group (Fig. 1B, Table 2). As revealed by HE staining, the decidual tissue of mice in the URSA group was sparsely structured and unevenly stained, with larger areas of hemorrhage and necrosis, and the structure of the necrotic cells, as well as the borders between the necrotic cells and the decidual tissues, was not clear (Fig. 1C), which suggested that the modeling was a success.

**Table 2:** The embryo resorption rate of mice in each group

Groups	Embryo resorption rate (%)									
	n1	n2	n3	n4	n5	n6	n7	n8	n9	n10
Normal	2.51	2.83	3.89	3.54	2.53	2.13	4.16	4.02	3.68	3.29
URSA	48.51	27.74	38.65	34.26	29.04	14.21	23.57	26.92	31.83	36.91
URSA + NS	32.52	39.72	24.34	39.54	26.72	34.21	30.26	33.33	38.57	15.59
URSA + Daphnetin (1 mg/kg)	26.09	25.64	24.87	20.31	38.01	31.02	26.15	27.49	22.78	16.94
URSA + Daphnetin (10 mg/kg)	24.09	23.56	16.1	25.58	12.27	16.75	22.47	14.61	13.82	17.02
URSA + Daphnetin (20 mg/kg)	13.45	18.74	25.79	12.57	26.47	20.39	29.07	24.5	25.03	27.96
sh-NC + URSA + Daphnetin (10 mg/kg)	18.49	12.95	24.45	16.21	21.86	22.74	17.62	18.04	11.63	19.59
sh-NR4A1 + URSA + Daphnetin (10 mg/kg)	25.65	29.51	22.28	18.46	31.52	23.76	34.26	39.81	29.73	26.21
sh-NR4A1 + oe-NC + URSA + Daphnetin (10 mg/kg)	38.57	30.94	30.82	28.67	21.06	19.36	29.31	21.69	25.73	35.26
sh-NR4A1 + oe-BACH2 + URSA + Daphnetin (10 mg/kg)	18.54	19.25	17.46	27.05	14.81	13.76	20.71	26.52	22.64	18.97

URSA, unexplained recurrent spontaneous abortion; NS, normal saline; sh, short hairpin RNA; NC, negative control; NR4A1, nuclear receptor subfamily 4immunitygroup A member 1; BACH2, BTB and CNC homolog 2

Subsequently, mice with URSA were administered daphnetin at low, medium, and high doses (1 mg/kg, 10 mg/kg, and 20 mg/kg) via gavage. Mice treated with daphnetin had fewer immature, resorbed, or dead embryos compared to the mice treated with NS (Fig. 1D). Daphnetin at 10 mg/kg showed the most pronounced therapeutic effect, and the most significant decrease in the rate of embryo resorption was noted in mice treated with 10 mg/kg of daphnetin (Fig. 1E). Therefore, the decidual tissues from mice treated with 10 mg/kg of daphnetin were subjected to subsequent experiments.

As shown by HE staining in Fig. 1F, daphnetin treatment at 10 mg/kg resulted in normal morphology of the decidual tissues and alleviated hemorrhage and necrosis. IGFBP-1 and PRL are markers of decidualization, and their diminished expression is closely associated with progression in URSA. Immunohistochemistry showed that the expression of IGFBP-1 and PRL was reduced in the decidual tissues of mice with URSA, and Daphnetin treatment restored the expression of both proteins (Fig. 1G). Flow cytometry was used to analyze the Treg and Th17 cell populations in the spleens of mice. The URSA group had a lower percentage of Treg cells and a higher percentage of Th17 cells in the CD4^+^ T cell population. By contrast, daphnetin gavage contributed to a higher percentage of Treg cells and a lower percentage of Th17 cells in the CD4^+^ T cell population (Fig. 1H). Immunofluorescence staining (Fig. 1I) also revealed reduced infiltration of Tregs (CD25^+^FoxP3^+^) and increased infiltration of Th17 cells (CD4^+^IL-17A^+^) in the decidual tissues of URSA mice. Daphnetin treatment reduced Th17 cell infiltration while increasing Treg cell infiltration.

### Daphnetin regulates the viability and Th17/Treg cell homeostasis in CD4^+^ T cells

**Fig. 2:**
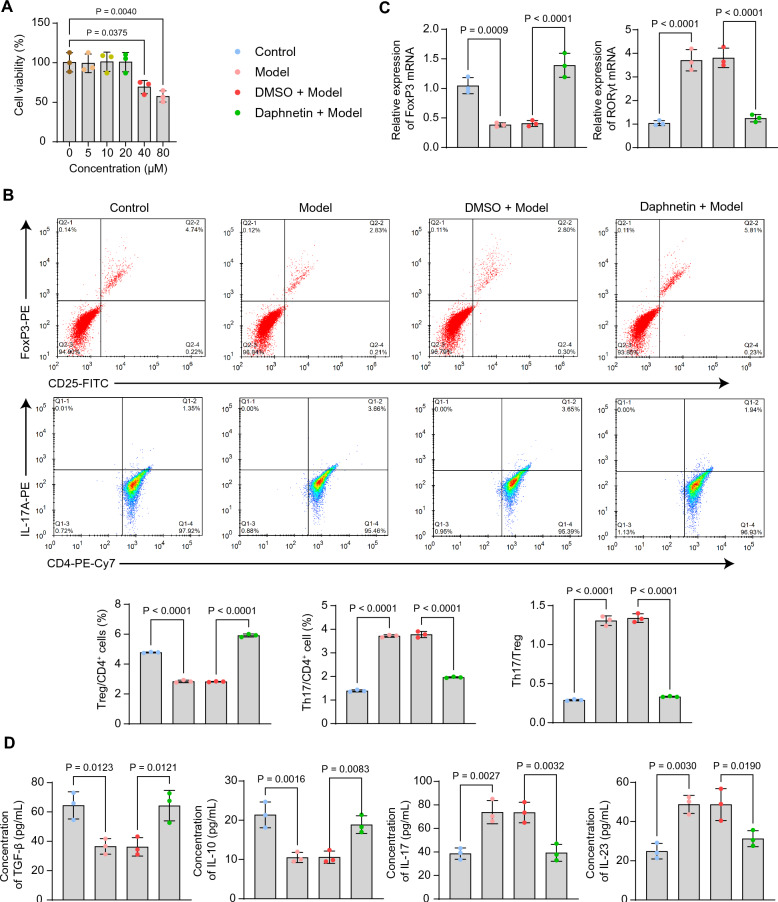
Daphnetin maintains Th17/Treg balance in CD4^+^ T cells. **A** Effect of different concentrations of daphnetin for 48 h on CD4^+^ T cell viability using the CCK-8 assay. **B** Percentage of Treg and Th17 cells in CD4^+^ T cells analyzed by flow cytometry. **C** Detection of mRNA expression of FoxP3 and RORγt in CD4^+^ T cells by RT-qPCR. **D** Concentrations of Treg cell-associated cytokines (TGF-β, IL-10) and Th17 cell-associated cytokines (IL-17, IL-23) in the supernatant of CD4^+^ T cells were determined by ELISA. Data is presented as mean ± SEM of three independent experiments. One-way ANOVA for ABCD

To verify the effect of daphnetin in vitro, we first treated CD4^+^ T cells with daphnetin at different concentrations (0, 5, 10, 20, 40, 80 μM) to observe the viability changes using CCK-8. The results showed that no remarkable toxicity was noted when the concentrations were lower than 20 μM, and daphnetin significantly decreased CD4^+^ T cell viability at concentrations higher than 40 μM (Fig. 2A). A decrease in the percentage of Treg cells and an elevation in the percentage of Th17 cells were observed in the cells of the model group activated with monensin, PMA, and ionomycin, which were reversed by daphnetin treatment (Fig. 2B). We further investigated the mRNA expression of FoxP3 and RORγt. The expression of FoxP3 was significantly reduced (*p* = 0.0009), and the expression of RORγt was significantly elevated (*p* < 0.0001) in the CD4^+^ T cells. The addition of daphnetin enhanced the expression of FoxP3 and suppressed the expression of RORγt (both *p* < 0.0001) (Fig. 2C). The concentrations of Treg cell-related cytokines and Th17 cell-related cytokines in CD4^+^ T cell supernatants were determined by ELISA. As expected, the levels of TGF-β (*p* = 0.0123) and IL-10 (*p* = 0.0016) were repressed, and the levels of IL-17 (*p* = 0.0027) and IL-23 (*p* = 0.0030) were augmented in the cells of the Model group. Daphnetin treatment, by contrast, elevated the levels of TGF-β (*p* = 0.0121) and IL-10 (*p* = 0.0083) and decreased the levels of IL-17 (*p* = 0.0032) and IL-23 (*p* = 0.0190) (Fig. 2D).

### Daphnetin upregulates the expression of NR4A1

**Fig. 3:**
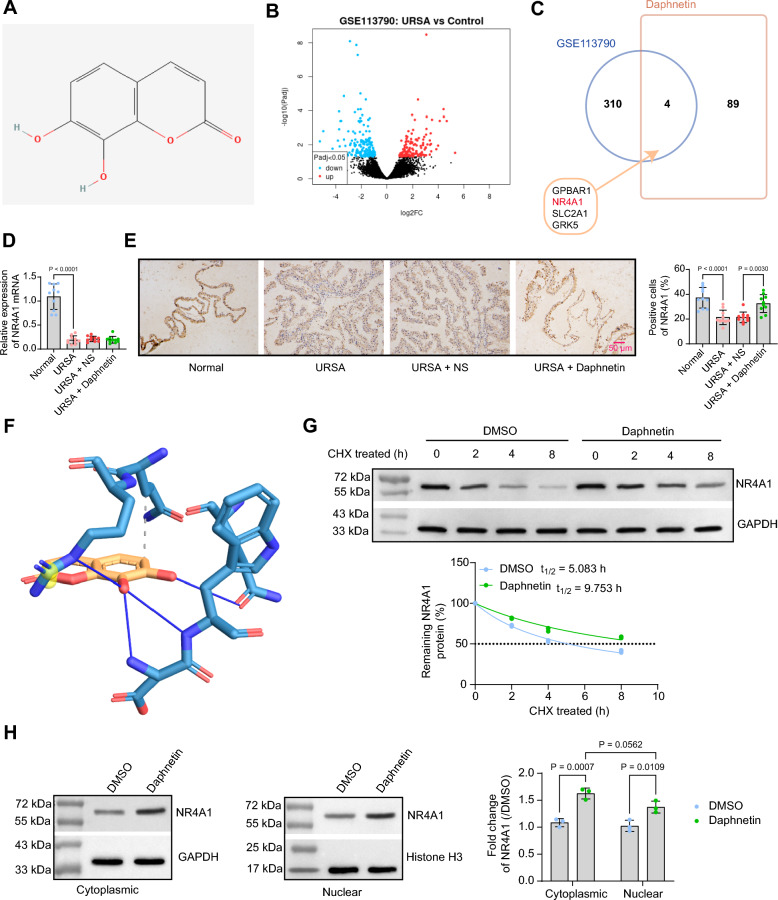
Daphnetin upregulates the protein expression of NR4A1. **A** Chemical structure of daphnetin from PubChem Substance. **B** The differentially expressed genes between decidual tissues from URSA patients and controls with induced abortions in the GSE113790 dataset. **C** Intersection of differentially expressed genes in the GSE113790 dataset and daphnetin downstream targets. **D** Detection of NR4A1 expression in mouse decidual tissue by RT-qPCR. **E** Positive cells of NR4A1 in mouse decidual tissue were detected by immunohistochemistry. **F** The molecular interaction between daphnetin and NR4A1 protein. **G** The residual intracellular NR4A1 protein expression in CD4^+^ T cells with 20 μM daphnetin and 10 μg/mL CHX for varying durations was measured using Western blot analysis. **H** NR4A1 expression in the nuclei and cytoplasm of CD4^+^ T cells treated with 20 μM daphnetin for 48 h was measured using Western blot analysis. Data is presented as mean ± SEM (n = 10 or 3). One-way ANOVA for DE, and two-way ANOVA for G

We obtained the chemical structure of Daphnetin (Fig. 3A) from PubChem Substance (https://www.ncbi.nlm.nih.gov/pcsubstance/) and predicted the molecular targets of daphnetin by Super-PRED (https:/ /prediction.charite.de/index.php). Next, the screening conditions of Benjamini & Hochberg (False discovery rate) with a significance level cut-off < 0.05 were used to analyze the RNA sequencing between the decidua tissues from URSA patients and controls with induced abortions in the GSE113790 dataset (Fig. 3B). Then, the differentially expressed genes in the GSE113790 dataset were intersected with downstream targets of daphnetin on Jvenn (https://jvenn.toulouse.inrae.fr/app/example.html). Four intersections (Fig. 3C): GPBAR1, (Log2FoldChange = 1.95), NR4A1 (Log2FoldChange = −2.65), SLC2A1 (Log2FoldChange = −2.02), and GRK5 (Log2FoldChange = −1.25) were obtained. We chose NR4A1, which had the most significant change in differential expression, for further study. The expression of NR4A1 in mouse decidual tissue was evaluated by RT-qPCR (Fig. 3D) and immunohistochemistry (Fig. 3E). Compared with the normal group, the mRNA expression and positive cell rate of NR4A1 were lower in the decidual tissue of mice in the URSA group. In contrast to the URSA + NS group, there was an insignificant change in the NR4A1 mRNA expression in the decidual tissues of mice in the URSA + Daphnetin group, whereas the rate of positive cells for NR4A1 was significantly higher.

We performed protein–ligand docking of daphnetin with the NR4A1 protein (PDB ID: 2QW4, NR4A1 ligand-binding domain) using HADDOCK 2.4 (https://rascar.science.uu.nl/haddock2.4/), revealing a binding relationship between the two (Fig. 3F). In human CD4^+^ cells, daphnetin treatment significantly prolonged the half-life (t1/2) of NR4A1 protein in CHX-treated cells, demonstrating enhanced NR4A1 protein stability (Fig. 3G). Additionally, daphnetin caused a significant increase in NR4A1 expression in both the nucleus and cytoplasm, with no significant difference in the fold change of NR4A1 expression between the two subcellular compartments, indicating that daphnetin does not affect the subcellular localization of NR4A1 (Fig. 3H). In summary, daphnetin enhances the expression of NR4A1 by binding to and increasing the stability of the NR4A1 protein.

### Downregulation of NR4A1 disturbs Th17/Treg cell homeostasis in mice with URSA

**Fig. 4:**
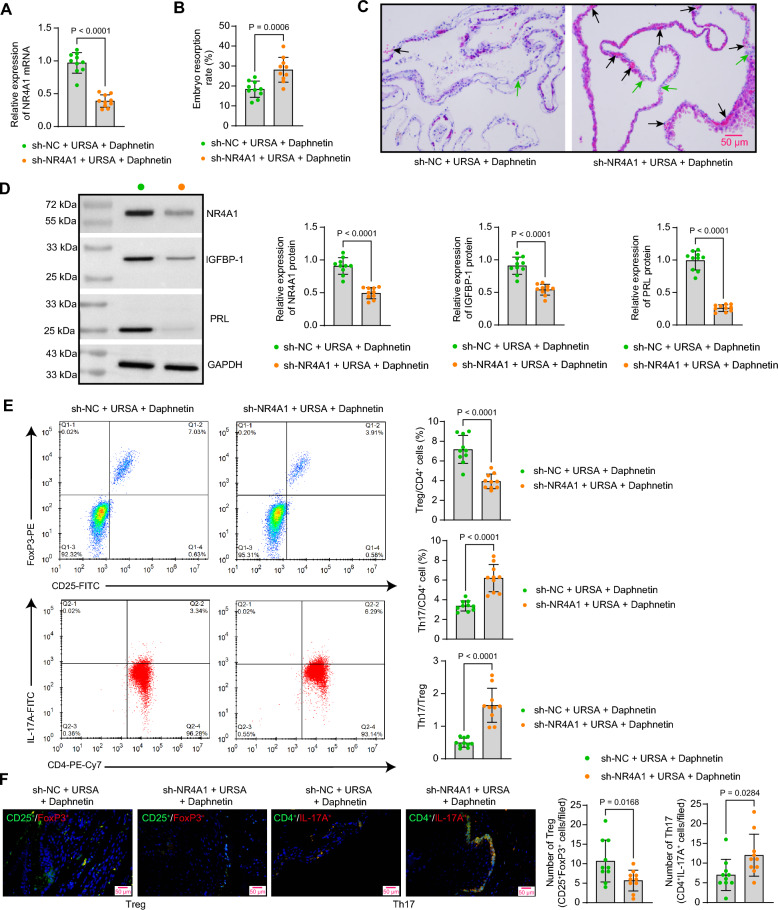
Knockdown of NR4A1 abates the mitigating effects of daphnetin on URSA in mice. **A** Knockdown efficiency of NR4A1 in the decidual tissue of URSA mice detected by RT-qPCR. **B** Embryo resorption rate in URSA mice after knockdown of NR4A1. **C** Morphologic changes (the black arrow indicates the site of hemorrhage, while the green arrow indicates the area of necrosis) in decidual tissues of URSA mice after the knockdown of NR4A1 were assessed by HE staining. **D** The protein expression of NR4A1, IGFBP-1, and PRL in decidual tissues of mice after the knockdown of NR4A1 was detected by western blot analysis. **E** Treg and Th17 cell populations in the spleens of mice were analyzed using flow cytometry. **F** The number of Tregs (CD25^+^FoxP3^+^) and Th17 cells (CD4^+^IL-17A^+^) infiltrating the decidual tissues of mice was assessed by immunofluorescence staining. Data is presented as mean ± SEM (n = 10). Unpaired t-test for ABDEF

URSA mice were pre-infected with adeno-associated viruses to knock down NR4A1 expression, followed by Daphnetin treatment. The knockdown efficiency of NR4A1 in the decidual tissues was substantiated using RT-qPCR (Fig. 4A). The embryo resorption rate of mice was increased after the knockdown of NR4A1 (Fig. 4B, Table 2). HE staining showed that the decidua of mice in the sh-NR4A1 + URSA + Daphnetin group was sparsely structured, and the areas of hemorrhage and necrosis were enlarged (Fig. 4C). Protein expression of NR4A1, IGFBP-1, and PRL was reduced in the decidual tissue of URSA mice after sh-NR4A1 intervention by western blot assay (Fig. 4D). In addition, Treg and Th17 cell populations in the spleens of mice after the knockdown of NR4A1 were analyzed. The proportion of Treg cells was significantly reduced, while the proportion of Th17 cells in the CD4^+^ T cell population was elevated following sh-NR4A1 treatment (Fig. 4E). Immunofluorescence staining revealed that NR4A1 knockdown resulted in a decrease in Tregs and an increase in Th17 cells within the decidual tissues (Fig. 4F).

### BACH2 is a target of the transcription factor NR4A1

**Fig. 5:**
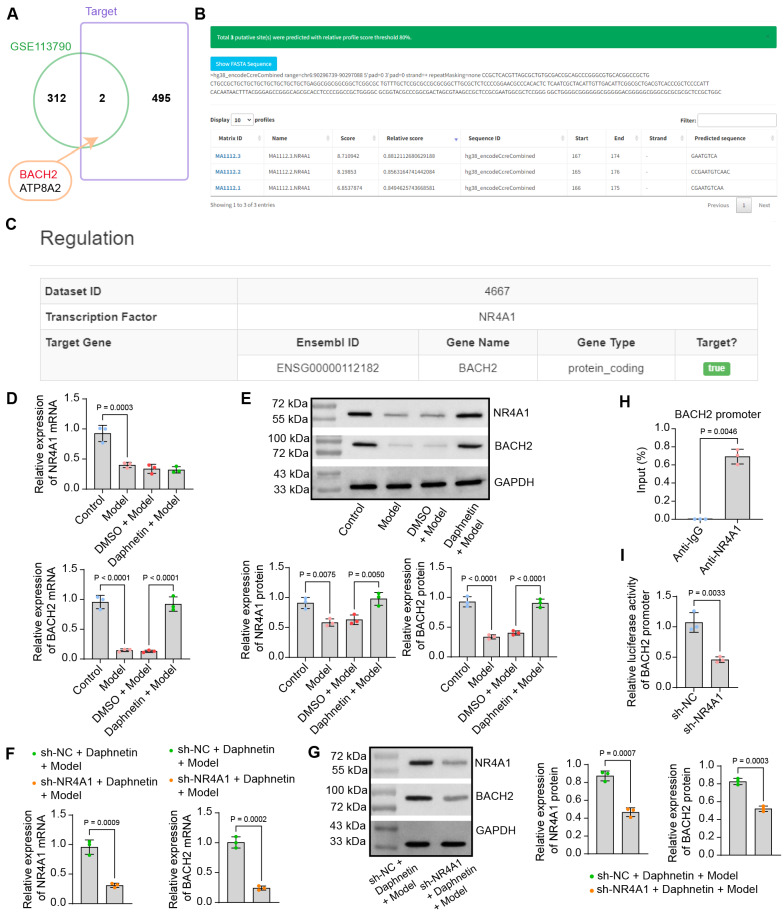
NR4A1 induces the transcription of BACH2 in CD4^+^ T cells. **A** The intersection of the first 500 downstream targets of NR4A1 downloaded from hTFtarget with differentially expressed genes in the GSE113790 dataset. **B** Binding sites for NR4A1 present in the promoter region of BACH2 (chr6: 90296739–90297088) were analyzed by JASPAR. **C** BACH2 was identified as a downstream target of NR4A1 in the hTFtarget database. **D** Detection of NR4A1 and BACH2 mRNA expression in CD4^+^ T cells by RT-qPCR. **E** Detection of NR4A1 and BACH2 protein expression in CD4^+^ T cells by western blot analysis. Expression of NR4A1 and BACH2 in CD4^+^ T cells after knockdown of NR4A1 was detected by RT-qPCR **F** and western blot **G**. **H** Enrichment of the BACH2 promoter immunoprecipitated by anti-NR4A1 in CD4^+^ T cells was analyzed using ChIP-qPCR. **I** Luciferase activity of the BACH2 promoter luciferase reporter plasmid in CD4^+^ T cells following NR4A1 knockdown was detected by dual-luciferase assays. Data is presented as mean ± SEM of three independent experiments. Unpaired t-test for FGHI, and one-way ANOVA for DE

To further explore the downstream mechanisms of NR4A1 in URSA, we downloaded the top 500 downstream targets of NR4A1 from hTFtarget (https://guolab.wchscu.cn/hTFtarget/#! /) and obtained 2 intersections (Fig. 5A) with differentially expressed genes in the GSE113790 dataset on Jvenn: BACH2 (Log2FoldChange = −1.94) and ATP8A2 (Log2FoldChange = −1.90). BACH2, which has more significant differential expression changes, was selected for our following study. Analysis using JASPAR (https://jaspar.elixir.no/) revealed an NR4A1 binding sequence (CCGAATGTCAAC) located between 165 and 176 bp within the promoter region of BACH2 (chr6: 90296739–90297088) (Fig. 5B), which was also corroborated by the regulation relationship on the hTFtarget website (Fig. 5C).

The expression of NR4A1 and BACH2 in CD4^+^ T cells was detected by RT-qPCR (Fig. 5D) and western blot analysis (Fig. 5E). The expression of both NR4A1 and BACH2 was low in the induced CD4^+^ T cells. Daphnetin treatment restored the expression of BACH2 and NR4A1 protein, while the mRNA expression of NR4A1 was not significantly changed. Infection of CD4^+^ T cells using lentivirus harboring sh-NR4A1 revealed that NR4A1 silencing caused the NR4A1 and BACH2 downregulation in the presence of modeling and daphnetin treatments (Fig. 5F,G). In addition, ChIP assay results presented that the BACH2 promoter was significantly enriched by anti-NR4A1 in CD4^+^ T cells (Fig. 5H). Moreover, sh-NR4A1 significantly reduced the relative luciferase activity of the BACH2 promoter luciferase reporter plasmid in CD4^+^ T cells (F5g. 5I).

### Overexpression of BACH2 maintains Th17/Treg cell homeostasis in CD4^+^ T cells in the presence of sh-NR4A1

**Fig. 6:**
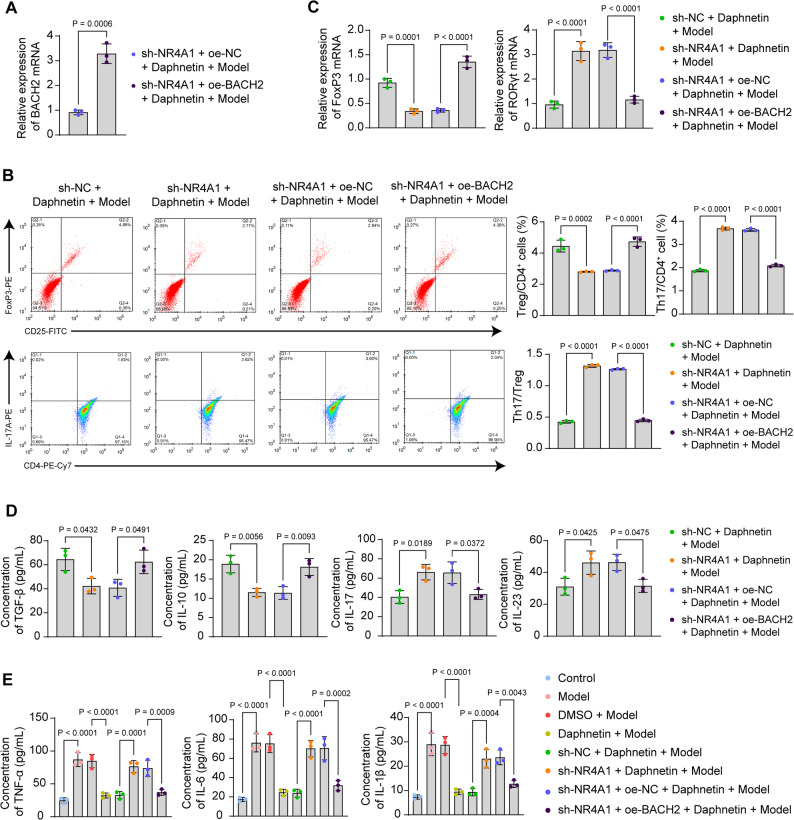
Overexpression of BACH2 is beneficial in maintaining Th17/Treg cell homeostasis in CD4^+^ T cells in the presence of sh-NR4A1. **A** Overexpression efficiency of BACH2 in CD4^+^ T cells detected by RT-qPCR. **B** Percentage of Treg and Th17 cells in CD4^+^ T cells analyzed by flow cytometry. **C** Detection of mRNA expression of FoxP3 and RORγt in CD4^+^ T cells by RT-qPCR. **D** Concentrations of Treg cell-associated cytokines (TGF-β, IL-10) and Th17 cell-associated cytokines (IL-17, IL-23) in the supernatant of CD4^+^ T cells were determined by ELISA. **E** The levels of pro-inflammatory factors TNF-α, IL-6, and IL-1β in CD4^+^ T cells were measured by ELISA. Data is presented as mean ± SEM of three independent experiments. Unpaired t-test for A, one-way ANOVA for BCDE

CD4^+^ T cells were infected with lentiviruses of sh-NR4A1 and oe-BACH2, and the overexpression efficiency of BACH2 was detected by RT-qPCR (Fig. 6A). Analysis by flow cytometry showed a significant decline in the percentage of Treg cells and a restoration in the percentage of Th17 cells in the CD4^+^ T cells infected with sole sh-NR4A1, which was reversed by the oe-BACH2 intervention (Fig. 6B). Knockdown of NR4A1 was found to inhibit mRNA expression of FoxP3 and promote mRNA expression of RORγt in CD4^+^ T cells by RT-qPCR assay. Overexpression of BACH2 restored the expression of FoxP3 and suppressed the expression of RORγt (Fig. 6C). Consistent results were obtained from ELISA to detect the concentrations of Treg cell-related cytokines (TGF-β, IL-10) and Th17 cell-related cytokines (IL-17, IL-23) in cell supernatants (Fig. 6D). The contents of classic pro-inflammatory cytokines TNF-α, IL-6, and IL-1β were also evaluated in CD4^+^ T cells using ELISA kits. The anti-inflammatory properties of daphnetin were found to be overturned by NR4A1 knockdown. Overexpression of BACH2 inhibited the pro-inflammatory cytokine contents (Fig. 6E).

### Upregulation of NR4A1-mediated BACH2 improves RSA in mice

**Fig. 7:**
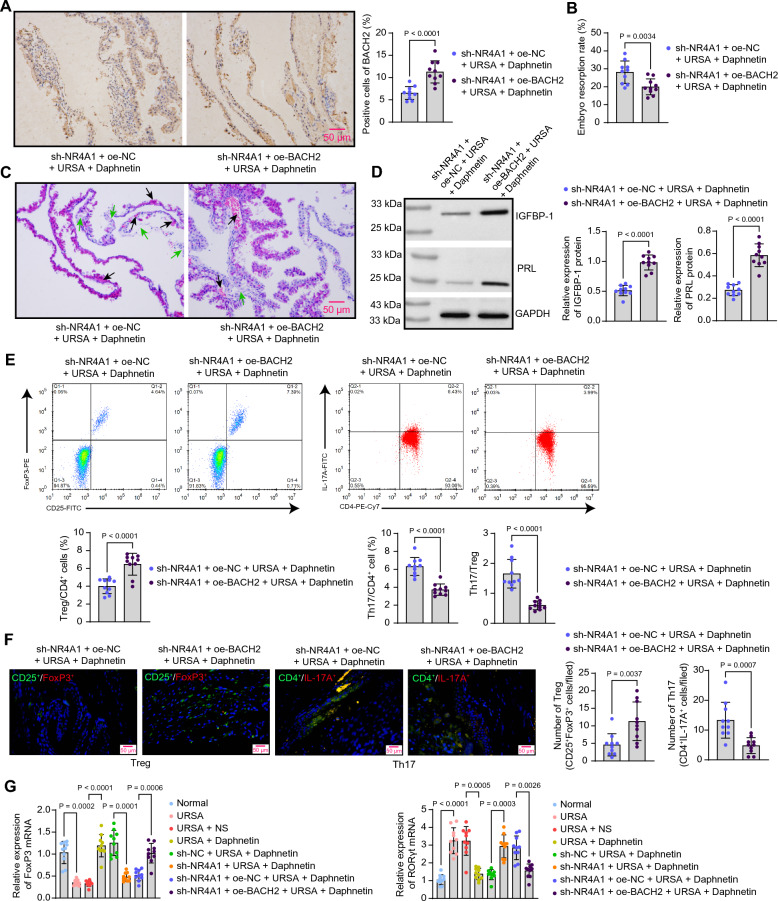
Overexpression of BACH2 overturns the accentuating effects of sh-NR4A1 on URSA in mice. **A** Overexpression efficiency of BACH2 in the decidual tissue of URSA mice detected by immunohistochemistry. **B** Embryo resorption rate in URSA mice after knockdown of NR4A1 and overexpression of BACH2. **C** Morphologic changes (the black arrow indicates the site of hemorrhage, while the green arrow indicates the area of necrosis) in decidual tissues of URSA mice after the knockdown of NR4A1 and overexpression of BACH2 were assessed by HE staining. **D** The protein expression of IGFBP-1 and PRL in decidual tissues of mice after the knockdown of NR4A1 and overexpression of BACH2 was detected by western blot analysis. **E** Treg and Th17 cell populations in the spleens of mice were analyzed using flow cytometry. **F** The number of Tregs (CD25^+^FoxP3 +) and Th17 cells (CD4^+^IL-17A^+^) infiltrating the decidual tissues of mice was assessed by immunofluorescence staining. **G** mRNA expression of FoxP3 and RORγt in decidual tissue of URSA mice by RT-qPCR. Data is presented as mean ± SEM (n = 10). Unpaired t-test for ABDEF, and one-way ANOVA for G

In the same vein, we developed a batch of mice pre-infected with adeno-associated viruses of sh-NR4A1 + oe-BACH2 or sh-NR4A1 + oe-NC before modeling and daphnetin treatment. The overexpression efficiency of BACH2 in the decidua of mice pre-treated with oe-BACH2 was detected by immunohistochemistry (Fig. 7A). The embryo resorption rate was significantly lower in mice overexpressing BACH2 (Fig. 7B, Table 2). As shown by HE staining, the morphology of the decidual tissue of mice overexpressing BACH2 returned to normal, and the degree of hemorrhage and necrosis was alleviated (Fig. 7C). Western blot assays revealed that the IGFBP-1 and PRL protein expression was elevated in the decidual tissue of mice pre-infected with sh-NR4A1 and oe-BACH2 (Fig. 7D). Th17/Treg cell homeostasis was restored by overexpression of BACH2, as evidenced by Treg and Th17 cell populations in the spleens and decidual tissues of mice (Fig. 7E, F) and the mRNA expression of FoxP3 and RORγt in the decidual tissue of mice (Fig. 7G).

## Discussion

Because of elusive etiology and mechanisms, clinical management is exceedingly challenging, and progress in reproductive immunology might offer hope for developing tailored treatment strategies [14]. In this study, the homeostasis of Treg and Th17 cells in the spleen and decidual tissues was disrupted in mice with URSA, which was recovered by daphnetin treatment; thus, clarifying the detailed mechanisms is essential for advancing the application of daphnetin in the management of URSA.

The anti-inflammatory properties of daphnetin have been shown in various conditions, including silica-induced pulmonary inflammation [15], lipopolysaccharide-induced lung injury [16], and corneal alkali burn [17]. Therefore, we sought to describe the detailed molecular mechanism involved. Daphnetin has been found to ameliorate neuropathic pain via the regulation of microglial responses [18]. Moreover, daphnetin has been revealed to significantly alleviate erythema, scaling, epidermal thickness, and inflammatory cell infiltration in psoriasis-like mice by attenuating upregulation of IL-6, IL-23A, and IL-17A in the skin lesions of mice [19]. These findings suggested the possible immunoregulatory function of daphnetin. More specifically, daphnetin ameliorated experimental colitis by Treg/Th17 balance [20]. Considering the increasing importance of the Treg/Th17 balance in the development of URSA [21, 22], we sought to explore whether daphnetin had a similar role in URSA. As expected, the mice with URSA showed higher embryo resorption rates and reduced positive rates of IGFBP-1 (which plays a chief role in the process of implantation) [23] and PRL (a pleiotropic hormone with immunomodulatory functions mainly related to reproduction) [24]. Daphnetin at 10 mg/kg was found to restore Treg and repress the Th17 cell population. Treg and Th17 cell differentiation is induced from naive precursors, during which TGF-β induces FoxP3 expression, while IL-6-mediated activation of STAT3 is required for RORγt expression [25]. Here, the proportion of Treg, the concentrations of TGF-β and IL-10, as well as the mRNA expression of FoxP3 in CD4^+^ T cells, were reduced by the modeling process and enhanced by daphnetin. By contrast, the induced Th17 cell population, the contents of IL-17 and IL-23, and RORγt mRNA expression were repressed by daphnetin.

Here, we revealed the direct interaction of daphnetin and NR4A1 using molecular docking and found that daphnetin prolonged the half-life of NR4A1 in human CD4^+^ T cells. Since they were originally cloned more than 30 years ago, the NR4A family has been implicated in many aspects of immune tolerance, including peripheral T cell tolerance and Treg [26]. For instance, NR4A1 has been revealed to be required for the effect of isoallolithocholic acid on Tregs, thereby maintaining immune homeostasis in humans [27]. Sekiya et al*.* showed that activation of NR4A1 via its agonist partially induced Treg cell differentiation [28]. NR4A1 expression in peripheral blood mononuclear cells was low during the preclinical stage of multiple sclerosis, and INF-γ and IL-17 production by proinflammatory Th1/Th17 cells in the central nervous system was decreased after NR4A1 agonist cytosporone B treatment [29]. These findings indicated that NR4A1 can modulate the Treg/Th17 cell balance. To be noted, our in vivo observation also showed that knockdown of NR4A1 led to elevated embryo resorption rates in mice induced with URSA, which also showed a higher Th17 cell proportion. In addition, we identified BACH2 as a downstream target of NR4A1 that was reduced in the decidual tissues of patients with URSA in the GSE113790 dataset. BACH2 (located on chromosome 6q15) has been implicated in the pathogenesis of several autoimmune diseases, and its expression has been identified in B and T cells [30]. For instance, BACH2 knockdown in CD4^+^ T cells induced the CD19^+^ B-cell count and secretion of IgG by prompting the release of cytokines [31]. The Treg-modulating factor BACH2 is indispensable for the inhibition of lethal inflammation, and a correlation between BACH2 and FoxP3 levels was identified in multiple sclerosis and Hashimoto’s thyroiditis, both autoimmune diseases [32]. Sasikala et al*.* also showed that decreased BACH2 expression and BACH2^+^/CD4^+^ T lymphocytes were observed in patients with chronic pancreatitis, and BACH2 repression resulted in Th17 cell-mediated inflammation [33]. In this study, overexpression of BACH2 overturned the disturbing effects of NR4A1 knockdown on homeostasis of Th17/Treg cells, thereby alleviating URSA in mice.

## Conclusion

**Fig. 8:**
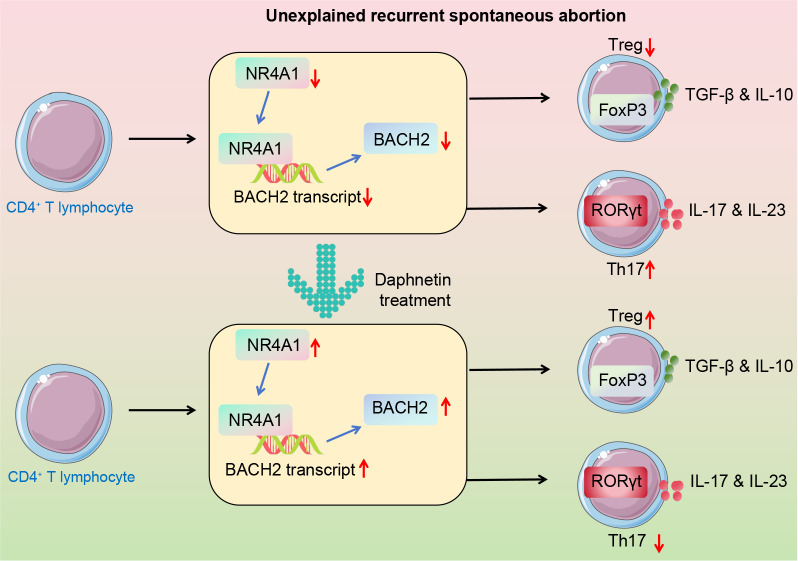
The protective effect of daphnetin on URSA and the underlying mechanisms. CD4^+^ T cells in URSA are polarized to Th17 differentiation, while Treg differentiation is reduced. Daphnetin ameliorated the imbalance of the Treg/Th17 ratio by promoting NR4A1-mediated BACH2 transcriptional expression, thereby inhibiting URSA progression

In conclusion, daphnetin prominently alleviated URSA by inducing the activation of the NR4A1/BACH2 axis and maintaining the homeostasis of Th17/Treg cells (Fig. 8). These findings provide novel insight into the management of URSA. While some coumarin derivatives have been reported to influence nuclear receptor activity [34, 35], specific data on NR4A1 are scarce. Further studies are necessary to confirm whether NR4A1 can be regulated by other coumarin derivatives.

## Data Availability

Data will be made available on request.
